# Analyzing the Effect of Factors on Individuals’ Subjective Well-Being with Quantile Bayesian Structural Equation Modeling Approach

**DOI:** 10.3390/bs14121170

**Published:** 2024-12-06

**Authors:** Zübeyde Çiçek, Nuran Bayram Arlı

**Affiliations:** 1 Department of Statistics, Faculty of Engineering and Natural Sciences, Suleyman Demirel University, Isparta 32200, Türkiye; 2 Department of Econometrics, Faculty of Economics and Administrative Sciences, Bursa Uludag University, Bursa 16059, Türkiye; nuranb@uludag.edu.tr

**Keywords:** subjective well-being, happiness, life satisfaction, Quantile Structural Equation Model

## Abstract

This study investigates factors influencing individuals’ levels of subjective well-being by examining fundamental variables, called life domains, such as satisfaction with health, education, marriage, housing district, work life, social life, relationships, and public services. The aim is to understand how these domains affect various quantile values of subjective well-being. To achieve this, the Life Satisfaction Survey dataset for 2020, obtained from the Turkish Statistical Institute, was utilized, and Bayesian Quantile Structural Equation Modeling and Bayesian Structural Equation Modeling methods were applied. The empirical study indicates that all life domains were found to have a positive impact on subjective well-being, except for housing-district satisfaction and education satisfaction. In particular, for individuals with low subjective well-being, satisfaction with work life, relationships, and public services has a stronger effect on subjective well-being compared to those in higher quantiles. Conversely, marriage satisfaction has a stronger effect on subjective well-being among individuals in the higher quantiles. This study’s unique contribution is that examining subjective well-being according to quantile values provides detailed information on how the factors influencing individuals’ subjective well-being vary across different levels.

## 1. Introduction

Studies on subjective well-being’s presence, causes, and connections are rapidly increasing, garnering significant interest across various disciplines. Major disciplines that investigate subjective well-being include psychology, political science, economics, health science, education science, and social sciences [1,2,3,4,5,6]. Research conducted in these disciplines focuses on different dimensions of subjective well-being, providing essential information to improve human well-being and to make effective decisions for policymakers. For example, while psychology research examines individuals’ emotional states and personal happiness levels and attempts to understand the psychological factors that affect subjective well-being, economics research analyzes the relationship between individuals’ income levels and subjective well-being [7,8,9,10]. The effect of physical and mental health on subjective well-being is investigated in the field of health [11,12]. Studies in education examine the contributions of individuals’ educational levels and educational systems to subjective well-being; research in social sciences analyzes the effects of social activities, social relations, and social structure on subjective well-being [1,13,14,15,16]. All these studies provide crucial data for understanding and improving subjective well-being. However, more research is needed to gain a more comprehensive and in-depth understanding of subjective well-being. Research on subjective well-being provides essential clues for developing strategies that play a critical role in increasing the overall welfare of societies, and the implementation of these strategies is crucial for enhancing societal well-being.

The literature defines subjective well-being, happiness, life satisfaction, and quality of life concepts in very different ways. In some sources, these concepts have the same meaning, while in others, they are defined as subcomponents of each other [14,17]. Although there are studies showing that the concept of subjective well-being is used instead of quality of life, the lack of a full consensus in the literature on this significant subject underscores its importance. However, Diener [4] states that subjective well-being is a broader phenomenon that includes quality of life. In this context, subjective well-being can be described as a multidimensional concept that provides for overall life satisfaction, positive and negative emotional experiences, and the general quality of life.

To explain life satisfaction, happiness, and subjective well-being, which are concepts that are often used interchangeably, individuals’ objective conditions or subjective satisfaction variables are considered. Many studies in the literature are based on the approach that life satisfaction is primarily determined by objective conditions such as income, health, age, and education [1,14,18] etc. These factors are objective elements that are not shaped by individuals’ perceptions. In contrast, the second approach bases its explanation on satisfaction in different domains of life. This approach argues that individuals’ satisfaction levels are based on their personal perceptions, expectations, and values. These elements express individuals’ satisfaction in different domains of life, such as health satisfaction, financial satisfaction, job satisfaction, and marriage satisfaction, and are considered subjectively [19,20,21,22,23] etc. One of the limited studies in this field, by van Praag, Frijters, and Ferrer-i-Carbonell [24], provides a comprehensive analysis of the factors influencing general satisfaction, using German panel data from the years 1992 to 1997. The authors investigate various life domains, such as health, financial, job, leisure, housing, and environmental satisfaction, to understand their impact on individual general satisfaction. Additionally, the effects of objective factors such as age, income, gender, and education on individuals’ satisfaction levels across different life domains have been examined. Similarly, Easterlin and Swanga [25] examine the relationship between happiness and satisfaction across various life domains, using data from the General Social Survey for the years 1973 and 1994 in the United States. Their research analyzes the effects of the objective conditions (age, education, etc.) that influence individuals’ happiness and satisfaction levels, along with satisfaction levels in various life areas, including financial situation, family life, job, and health, on overall happiness.

In the context of similar studies conducted in Türkiye, the Turkish Life Satisfaction Survey (TLSS) is the most widely utilized data source, including in the present study. This survey offers a comprehensive dataset for analyzing subjective well-being across various life domains. For instance, Köksal, Uçak, and Şahin [26] examined the impact of satisfaction in diverse life domains on happiness at the micro level, using data from the 2008–2012 TLSS. Their analysis consistently identified income, marriage, and health satisfaction as the top predictors of happiness over the years. Similarly, Eren and Aşıcı [27] employed TLSS data from 2004 to 2013 to explain happiness through both objective and subjective indicators, such as income and job satisfaction. More recently, Güzel and Görmüş [28] utilized data from the 2019 TLSS to investigate the relationship between happiness and various satisfaction indicators. Their findings revealed that all factors, with the exception of education satisfaction, had a positive impact on happiness, including satisfaction with health, marital life, income, and other domains. Furthermore, Çakmak and Emirhan [29] used microdata from the 2014–2017 TLSS to investigate overall life satisfaction through six different domains, highlighting the significant influence of financial and marriage satisfaction, while also analyzing the effects of objective indicators such as gender and education on satisfaction levels.

As previously noted, despite the interchangeable use of terms like happiness, overall life satisfaction, and subjective well-being, it is essential to elucidate the concept of subjective well-being, which is central to our study. Subjective well-being consists of three subcomponents: life satisfaction, positive affect, and negative affect. While life satisfaction constitutes the cognitive component, the positive-negative affect component constitutes the affective component [14,30]. Life satisfaction, the cognitive component, refers to how individuals evaluate their lives as a whole. The affective component addresses whether individuals experience positive or negative moods. On the other hand, quality of life is generally expressed as ‘objective’ and describes a person’s living conditions rather than their response to these conditions [4]. According to many studies conducted since the 1980s, subjective well-being has been shown to consist of a cognitive component (life satisfaction) and an affective factor (happiness) [18,19,30]. Happiness has various definitions in literature and popular culture. It can refer to a positive mood at any given moment, living a good life, a general evaluation of life satisfaction, or the reasons that make a person happy. Therefore, some researchers prefer words with more specific meanings instead of using this term in its simple form. On the other hand, life satisfaction shows how an individual evaluates their life as a whole [4,30].

Among these scales, those developed by Diener [31] and Emmons and Diener [30], which have been validated in different languages, stand out and are widely used in various studies (for example, see [32,33,34,35,36]). In addition, subjective well-being can be measured with general satisfaction and happiness questions, as used in the World Values Survey [37]. Therefore, many studies in the literature explain the concept of subjective well-being with the concepts of life satisfaction and happiness (for example, see [5,7,38,39]). For instance, Lin and Yeh [40] used the Satisfaction with Life Scale [41] and the Long Term Affect Scale [42] in their study to measure well-being. In this context, measuring subjective well-being requires considering a latent variable structure. Therefore, the use of Structural Equation Modeling (SEM) instead of classical regression models has become more popular in the literature over time in explaining subjective well-being. Whether classical regression or SEM, results are obtained through the mean value. On the other hand, researchers use quantile regression analysis to measure the effects of explanatory variables at different quantile levels of subjective well-being. For example, as stated in Binder and Coad [43], income, health, social index, and education variables have been found to have a more substantial effect on life satisfaction at lower quantile levels. Similarly, Yuan and Golpelwar [44] tested the effects of the four domains of Social Quality—socioeconomic security, social inclusion, social cohesion, and social empowerment—on subjective well-being at different quantile values. Additionally, Neira et al. [39] examined the effects of different dimensions of social capital on individual-level subjective well-being.

However, although the quantile regression approach has found its place in the literature, it is essential to consider the SEM structure in this context. Indeed, a new group of studies in the psychology literature has started to use the quantile approach within the SEM framework, although in different fields. For example, in the study conducted by Zhang and Tang [45] using the World Values Survey data, they interpreted the latent variables of job satisfaction and religious belief, which are thought to affect life satisfaction, for different quantile values of life satisfaction. Similarly, in the study by Yazdi et al. [46], they used the Bayesian Quantile Structural Equation Modeling (BQSEM) method to analyze a psychological problems profile obtained through scales containing latent variables. They examined the effects of socioeconomic and personal stress on the psychological problems profile at different quantile values.

In studies examining the impact of different satisfaction domains on subjective well-being, general life satisfaction, or happiness, regression, logit, or probit models are typically employed for statistical analysis [2,24,25,26,27,28,29]. The existing literature has largely focused on investigating the effects of individual life domains on subjective well-being, life satisfaction, or happiness separately. In contrast, our research takes a different approach by utilizing latent variables to group similar factors, allowing for an examination of their combined effect on subjective well-being. To extend the literature, we employed a Quantile Structural Equation Model (QSEM) to assess how satisfaction in various domains affects subjective well-being across different quantile levels. This introduces a key question for discussion: How do the effects of different satisfaction domains on subjective well-being vary across each quantile level?

To address this, our study used life satisfaction and happiness variables from the Turkish Statistical Institute’s (TURKSTAT) 2020 Life Satisfaction Survey (LSS) dataset, making it the first study in the literature to apply the QSEM approach in this context. The findings offer valuable insights into the factors influencing individuals’ subjective well-being at various quantile levels and contribute to strategies for enhancing overall quality of life. The remainder of the study is organized as follows: the second section details the methodology, the third section presents and interprets the results from Bayesian Structural Equation Modeling (BSEM) and BQSEM using the WinBUGS (Windows version of Bayesian inference Using Gibbs Sampling) and *R* programs, and the final section concludes with a discussion and conclusion.

## 2. Materials and Methods

### 2.1. Study Sample

The purpose of the LSS, which has been regularly conducted by TURKSTAT every year since 2003, is to measure individuals’ perceptions of happiness and their general satisfaction levels in public and basic life areas [47]. This study utilizes the 2020 Türkiye LSS micro dataset obtained from TURKSTAT. Given the analysis of work life satisfaction, the estimates are restricted to working individuals. Additionally, only married individuals were included in the analysis for the marriage satisfaction variable. After performing necessary adjustments, the final sample consists of 2426 individuals.

### 2.2. Variable Description

The concept of subjective well-being is considered an endogenous variable in the study. The happiness (y_1_) and life satisfaction (y_2_) variables, which are included in the dataset of the LSS, constitute the latent variable “Subjective Well-Being” (η).

Latent variables and observed variables explaining these latent variables utilized in this study are presented in Table 1 with the questions in the survey.

**Figure 1:**
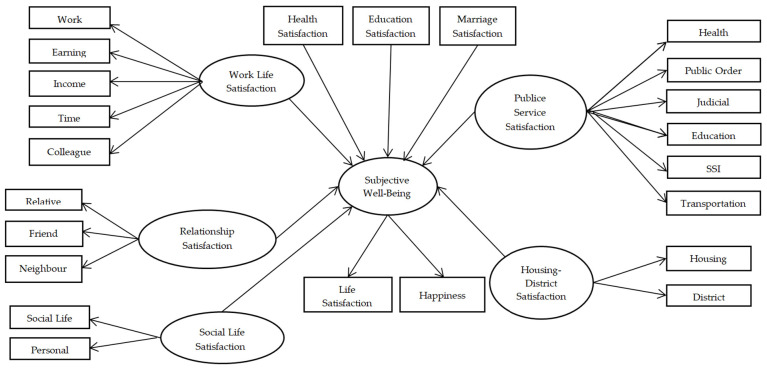
Research Model.

Housing (y_3_) and District (y_4_) satisfactions that are included in the LSS constitute the “Housing-District Satisfaction” (ξ_1_) exogenous variable, Work (y_5_), Earning (y_6_), Income (y_7_), Time (y_8_), and Colleague (y_9_) satisfactions constitute the “Work Life Satisfaction” (ξ_2_) exogenous variable, and Social Life (y_10_) and Personal Time (y_11_) satisfactions constitute the “Social Life Satisfaction” (ξ_3_) exogenous variable. In addition, the satisfaction of Relative (y_12_), Friend (y_13_), and Neighbor (y_14_) constitutes the “Relationship Satisfaction” (ξ_4_) exogenous variable; satisfaction with “Health” (y_15_), “Public Order” (y_16_), “Judicial” (y_17_), “Education” (y_18_), “Social Security Institution (SSI)” (y_19_), and “Transportation” (y_20_) constitute the “Public Service Satisfaction” (ξ_5_) exogenous variable. “Health Satisfaction” (d_1_), “Education Satisfaction” (d_2_), and “Marriage Satisfaction” (d_3_) have been added to the model as a covariate variable. The research model indicating the structure between latent variables is given in Figure 1.

This study examined the factors affecting individuals’ subjective well-being levels comprehensively. Subjective well-being is a concept that expresses individuals’ satisfaction with their lives and their general level of happiness. This study analyzed the effects of various life domains such as health satisfaction, education satisfaction, marriage satisfaction, housing-district satisfaction, work life satisfaction, social life satisfaction, relationship satisfaction, and public policy satisfaction on individuals’ subjective well-being. The following hypotheses form the basis of this study. They are tested to understand the effects of individuals’ satisfaction in different areas of their lives on subjective well-being.

**Hypothesis** **1** **(H_1_):** *There is a positive effect of health satisfaction on subjective well-being*.

**Hypothesis** **2** **(H_2_):** *There is a positive effect of education satisfaction on subjective well-being*.

**Hypothesis** **3** **(H_3_):** *There is a positive effect of marriage satisfaction on subjective well-being*.

**Hypothesis** **4** **(H_4_):** *There is a positive effect of housing-district satisfaction on subjective well-being*.

**Hypothesis** **5** **(H_5_):** *There is a positive effect of work life satisfaction on subjective well-being*.

**Hypothesis** **6** **(H_6_):** *There is a positive effect of social life satisfaction on subjective well-being*.

**Hypothesis** **7** **(H_7_):** *There is a positive effect of relationship satisfaction on subjective well-being*.

**Hypothesis** **8** **(H_8_):** *There is a positive effect of public policy satisfaction on subjective well-being*.

### 2.3. Statistical Methods

The classical SEM includes measurement equations to explain latent variables by utilizing more than one observed variable. Furthermore, structural equations which are essentially regression models based on the mean have been used to investigate how exogenous latent variables affect the outcomes of interest. In other words, the classical SEM does not merely provide a comprehensive analysis of the relationship between latent variables since it is built solely on the mean value. In QSEM, conditional quantile values of the endogenous latent variable are examined when exogenous latent variables and covariate variables are considered. Thus, the effect of exogenous variables can be compared for each level of the endogenous latent variable [48,49].

Where y_i_ = (y_i1_, …, y_ip_)^T^ (p × 1) represents the i observed variables vector with a sample size of n, and ω_i_ = (ω_i1_, …, ω_iq_)^T^ (q × 1) (q < p) represents the latent variables vector with a dimension, the measurement model showing the relationship between y_i_ and ω_i_ is as follows:

(1) $$y_{i}=Ac_{i}+\Lambda \omega_{i}+\varepsilon_{i}$$

Here, Α (p × r_1_) and Λ (p × q) are the unknown coefficients matrix, c_i_ (r_1_ × 1) is the fixed variables vector, and ε_i_ (p × 1) is the error terms vector. In Equation (1), ω_i_ = (η_i_^T^, ξ_i_^T^)^T^, η_i_ is the (q_1_ × 1) dimensional endogenous latent variables vector, and ξ_i_ is the (q_2_ × 1) dimensional exogenous latent variables vector. The primary objective of SEMs is to analyze the variability of η_i_ given the information contained in ξ_i_. In classical SEM, when η_i_, ξ_i_, and the control variable d_i_ (r_2_ × 1) are given, the conditional mean of η_i_ is as follows.

(2) $$E\left(\eta_{İ}|\xi_{i},\;d_{i}\right)=Βd_{i}+\Gamma \xi_{i}$$

Here, Β (q_1_ × r_2_) and Γ (q_1_ × q_2_) are the matrices of estimated unknown coefficients. This conditional mean does not provide a comprehensive description of the relationship between latent variables. To obtain a more comprehensive result, the conditional quantile values of η_i_ for different quantile (τ) values (0 < τ < 1) can be considered. The conditional quantile value θ_τ_(η_i_|ξ_i_, d_i_) is:

(3) $$\theta_{\tau }\left(\eta_{i}|\xi_{i},d_{i}\right)={Β}_{\tau }d_{i}+\Gamma_{\tau }\xi_{i}$$

Here, the values of Β_τ_ and Γ_τ_ are obtained differently for each quantile value. Thus, the structural model for QSEM is obtained as follows:

(4) $$\eta_{i}={Β}_{\tau }d_{i}+\Gamma_{\tau }\xi_{i}+\delta_{i}$$

The measurement model equation defining QSEM is provided in Equation (1), and the structural model is given in Equation (4). Unlike the classical SEM, the distribution of δ_i_ is not specified in QSEM. The only assumption in QSEM is that the τ-quantile of the δ_i_ value is 0 to ensure the validity of Equation (4). While the structural model varies for different quantile values, the measurement model is limited to the median value. This is because the primary purpose of the measurement model is to link the observed variables with the latent factors; thus, quantile regression is meaningless in this context [45,49,50].

In conclusion, while the measurement model in QSEM is limited to the median regression model, the structural model can be constructed for different quantile values. Thus, QSEM provides more comprehensive results than classical SEM. In this study, the Bayesian estimation method, frequently preferred in the literature, was used for QSEM estimation. The Bayesian estimations conducted in this study were performed using the methodology and estimation methods developed by Wang in 2016 (see [50]).

The measurement equation of the model established with the observed variables Y = (y_1_, y_2_, …, y_20_)^T^ of the research model shown in Figure 1 is $y_{i}={\Lambda \omega }_{i}+\varepsilon_{i}$. Here, ω_i_ = (η_1_, ξ_1_, ξ_2_, ξ_3_, ξ_4_, ξ_5_)^T^ represents the latent variable and Λ denotes the factor loading matrix. η represents the endogenous latent variable, ξ_1_–ξ_6_ represents the exogenous latent variables, and d_1_, d_2_ and d_3_ represent the control variables, the structural equation is as follows:

(5) $$\eta_{1}=b_{0\tau }+b_{1\tau }d_{i1}+b_{2\tau }d_{i2}+b_{3\tau }d_{i3}+\gamma_{1\tau }\xi_{i1}+\gamma_{2\tau }\xi_{i2}+\gamma_{3\tau }\xi_{i3}+\gamma_{4\tau }\xi_{i4}+\gamma_{5\tau }\xi_{i5}+\gamma_{6\tau }\xi_{i6}+\delta_{i}$$

**Figure 2:**
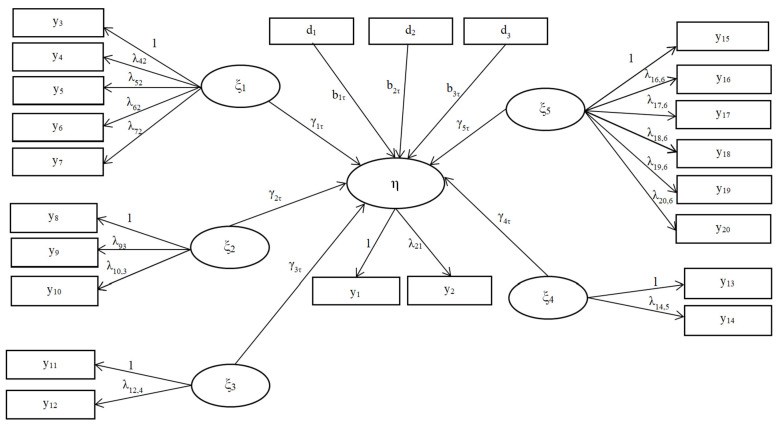
Symbolic representation of the path diagram on subjective well-being.

As a result, the research model given in Figure 1 is parametrized as shown in Figure 2 within the context of the structural equation provided in Equation (5).

Here, b_0*τ*_, b_1*τ*_, b_2*τ*_, b_3*τ*_, γ_1*τ*_, γ_2*τ*_, γ_3*τ*_, γ_4*τ*_, γ_5*τ*_, and γ_6*τ*_ show the unknown coefficients varying according to τ-quantile values (Figure 2). The quantile values are 5%, 10%, 25%, 50%, 75%, 90%, and 95%.

### 2.4. Prior Distribution

The prior distributions of the hyperparameters included in the models must be determined through Bayesian analyses. For BQSEM, the prior means of Λ_yk_ have a conjugate normal distribution with Λ_0yk_ = 1 ve H_0yk_ matrix with diagonal elements of 10,000. All values of prior means of Λ_ωk_ are taken as Λ_0ω_ = 1, and the H_0ω_ covariance matrix is also taken as 10,000 times the unit matrix. The other hyperparameters are α_0yk_ = α_0σ_ = 9 and β_0yk_ = β_0σ_ = 4 for the conjugate inverse Gamma distribution of σ_yk_ and σ_η_, and ρ_0_ = 6 and R_0_ = 15*I_5_ for the inverse Wishart distribution of Φ. Given the absence of models with latent structures in the Turkish literature on subjective well-being, the standard prior structure typically employed in BQSEM models was applied here to maintain methodological consistency and ensure reliable inference (see, [45,49]). Furthermore, robustness checks were conducted with alternative prior estimates, underscoring the stability and robustness of the chosen prior settings within this modeling framework.

### 2.5. Determination of Convergence

Convergence must be ensured in Bayesian estimation methods to test the consistency of the estimated parameters. Although various techniques are available for this purpose, trace and density plots were used in this study to evaluate Bayesian estimations of both QSEM and classical SEM.

**Figure 3:**
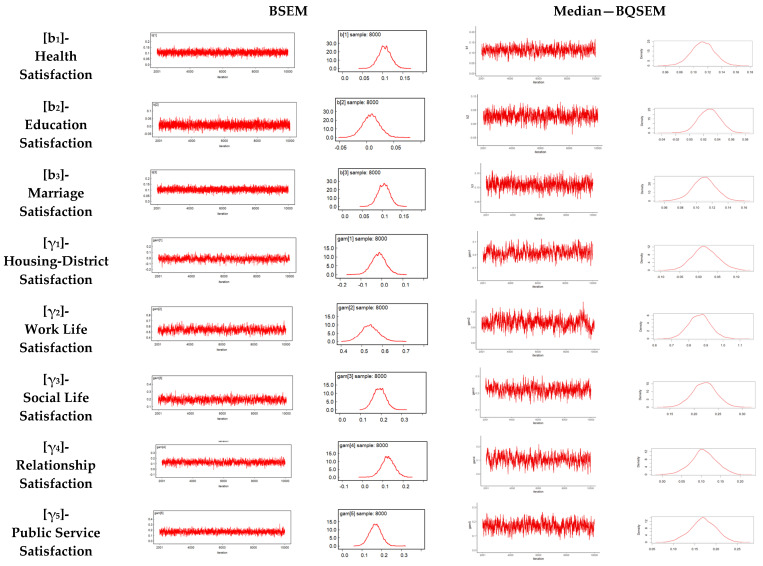
Trace and density plots of structural model parameters of BSEM and Median-BQSEM.

In the application, the posterior distribution was simulated using Gibbs sampling, one of the MCMC methods. Although there are different approaches for choosing the number of chains, a single chain with a high number of iterations was preferred over multiple chains. As a result of the trials for both models, it was observed that the model converged after 10,000 iterations. Thus, it was decided to perform 10,000 iterations for the MCMC simulation. In addition, it was observed that convergence is achieved, and the series becomes stationary, when the first 2000 observations are discarded with a burn-in period for both models. The trace and density plots of the parameters in the structural model after the burn-in period are shown in Figure 3. Only the plots for the median-BQSEM have been displayed, as the results for different quantile values in the BQSEM were very close.

Analyzing the trace and density plots of the estimated parameters obtained after the burn-in period in Median-BQSEM and BSEM shown in Figure 3, it can be seen that the structural model parameters converge to a certain value, and it is observed that all of them show normal distributions when examining the density plots. It was also concluded that the factor loadings for both models converged to a certain value and were typically normally distributed.

## 3. Results

The mean values, standard deviations, and correlation coefficients for the latent variables in the model are shown in Table 2. The correlation analyses demonstrate that subjective well-being is positively correlated with satisfaction in various life domains, including housing-district satisfaction, work life satisfaction, social life satisfaction, relationship satisfaction, and public service satisfaction. Furthermore, housing-district satisfaction is positively correlated with work life satisfaction, social life satisfaction, relationship satisfaction, and public service satisfaction. Similarly, work life satisfaction shows a positive correlation with social life satisfaction, relationship satisfaction, and public service satisfaction. Social life satisfaction is also positively correlated with relationship satisfaction and public service satisfaction, and relationship satisfaction is positively correlated with public service satisfaction.

At this stage of the study, classical BSEM and BQSEM were applied using similar prior distributions, iteration counts, burn-in periods, and chain numbers. Table 3 presents the unstandardized parameter estimates and credible intervals for BSEM and Median-BQSEM.

Table 3 shows the BQSEM prediction results for the median value (τ = 0.50) and the classical BSEM prediction results. The parameter γ_1_, representing the latent variable “Housing-District Satisfaction”, and the parameter b_3_, representing “Education Satisfaction”, are statistically insignificant in both models. All other domain satisfactions are statistically significant. The unstandardized values of the parameter estimates obtained for all quantiles in BQSEM are given in Table 4 together with the BSEM results.

Table 4 shows the factor loadings, structural model coefficients, and covariances between latent variables of BSEM and BQSEM for quantiles 0.05, 0.10, 0.25, 0.50, 0.75, 0.90, and 0.95. Since the posterior predictive *p*-value (PPP) goodness of fit values are around 0.664–0.675 in these seven quantile models, the models are found to be at an acceptable fit level.

As seen in Table 4, health satisfaction positively affects subjective well-being. No significant differences were observed across quantile levels. Additionally, marriage satisfaction has a positive effect on subjective well-being, with this effect being greater at higher levels of subjective well-being and smaller at lower levels. In contrast, the effects of housing-district satisfaction and educational satisfaction on subjective well-being were not found to be statistically significant in any of the BSEM or BQSEM models.

Satisfaction with work life was found to have a significant and positive effect on subjective well-being. When examined according to quantile levels, differences in the effect levels were observed. The effect of work life satisfaction on subjective well-being is higher for individuals with lower levels of subjective well-being, whereas this effect is lower for those with higher levels. Social life satisfaction also positively influences subjective well-being, as indicated by both BSEM and BQSEM results, where the coefficients obtained are quite close to each other, suggesting that satisfaction with social life affects subjective well-being at similar levels across quantile levels. Furthermore, relationship satisfaction was found to affect subjective well-being positively. It has been found that, at the 5%, 10%, and 25% quantile levels, meaning for those with low subjective well-being, relationship satisfaction affects their subjective well-being levels more. As the level of subjective well-being increases (quantile value increases), the effect of relationship satisfaction on subjective well-being decreases. Finally, satisfaction with public services positively affects subjective well-being, with its effect also decreasing as the quantile value increases, similar to relationship satisfaction.

**Figure 4:**
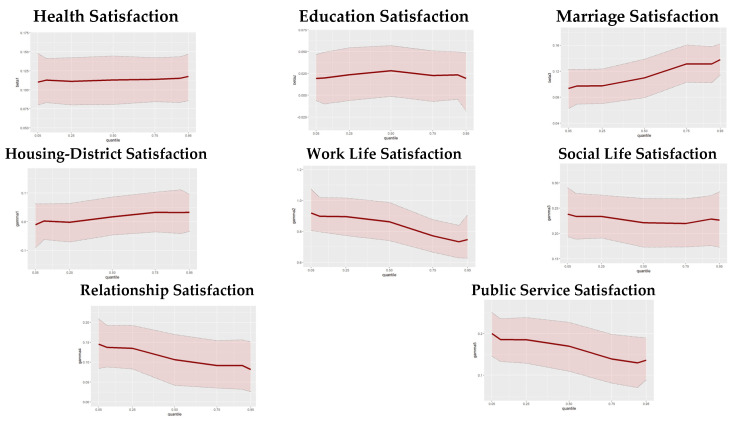
Graphs of structural model regression coefficients estimated for each quantile level and credible intervals at 95% confidence level.

The estimates of the parameters b_0τ_, b_1τ_, b_2τ_, b_3τ_, γ_1τ_, γ_2τ_, γ_3τ_, γ_4τ_, and γ_5τ_ obtained in the structural model according to each quantile value are given in Figure 4 with their credible intervals. Marriage satisfaction shows an upward trend, with a stronger effect at higher quantiles, while work life satisfaction, relationship satisfaction, and public services satisfaction show decreasing effects, indicating greater relevance for individuals at lower quantiles. In contrast, health satisfaction remains relatively stable with small fluctuations, while education satisfaction and housing-district satisfaction exhibit statistically insignificant effects across all quantiles. These patterns highlight the importance of quantile analyses, which show how certain domains affect subjective well-being differently across the distribution and provide deeper insights beyond mean-based models.

## 4. Discussion

In this study, the BSEM and BQSEM analyses examine the relationship between subjective well-being and different life domains, analyzing the factors affecting individuals’ subjective well-being in Türkiye. In addition, the analyses conducted for each quantile level examine how these satisfaction elements affect subjective well-being and the differences between individuals with low and high subjective well-being. The findings indicate that satisfaction with health, education, marriage, work life, social life, relationships, and public service positively affects subjective well-being. The main results obtained can be summarized as follows (Table 4):

Health satisfaction has a positive effect on subjective well-being and does not change significantly across quantile levels. In other words, individuals with both low and high levels of subjective well-being exhibit similar levels of health satisfaction. The stable relationship found here supports the notion that health satisfaction is a foundational element of overall happiness, reinforcing its critical role in promoting subjective well-being. Studies conducted in Türkiye also confirm this positive relationship between health satisfaction and both happiness and general satisfaction [26,28,29]. Moreover, findings from international literature further support this conclusion [24,25], underscoring the universal importance of health satisfaction in influencing subjective well-being.

The effect of education satisfaction on subjective well-being was found to be statistically insignificant in both the quantile model and the classical model. The literature shows mixed findings, with some studies identifying a strong relationship between education and subjective well-being, while others do not demonstrate a clear connection. Studies that have proven a positive relationship between education and subjective well-being include: [13,16,51,52,53,54,55]. In the national literature, however, both negative effects of education on happiness [56,57] and positive effects [27,58,59,60] have been observed. These mixed results suggest that, while education contributes to subjective well-being, its effect may be influenced by factors such as socioeconomic status, employment opportunities, and personal aspirations, which can vary greatly among different populations. While many studies focus on the direct effects of education level on happiness, Köksal, Uçak, and Görmüş [26], as well as Güzel and Görmüş [28], have similarly examined the education satisfaction variable in the context of Türkiye. Although Köksal, Uçak, and Görmüş [26] found a positive relationship between education satisfaction and happiness during the years 2010–2012, they did not find a significant effect in 2008 and 2009. On the other hand, Güzel and Görmüş [28] emphasized that there was no direct effect of education satisfaction on happiness in 2019.

Marriage satisfaction positively affects subjective well-being. As the level of subjective well-being increases, the effect of marriage satisfaction on subjective well-being also increases. In other words, for individuals with lower levels of subjective well-being, the impact of marriage satisfaction is weaker compared to those with higher levels. Conversely, for individuals with higher levels of subjective well-being, the contribution of marriage satisfaction to their overall well-being is stronger. Köksal, Uçak and Şahin, Güzel and Görmüş, and Çakmak and Emirhan, in their studies with Turkish samples from different years, emphasized that marriage satisfaction has a positive and significant effect on happiness, similar to the findings of this study [26,28,29]. These results support the idea that marriage satisfaction plays an important role in influencing individuals’ subjective well-being across various contexts and time periods.

The effect of housing-district satisfaction on subjective well-being was not found to be statistically significant. Although both national and international studies in the literature have shown a positive relationship between housing satisfaction and general satisfaction [24,27,28,29], no significant effect was found in this study. However, unlike those studies, this research examines the combined effect of housing satisfaction and district satisfaction as a latent variable on subjective well-being, offering a more comprehensive view of how these factors jointly influence well-being. This discrepancy may result from the fact that, when considering the influence of location, the relationship between housing-district satisfaction and subjective well-being can be affected by variables such as neighborhood quality, safety, and access to amenities.

It has been observed that individuals’ work life satisfaction positively affects their levels of subjective well-being. For those with lower levels of subjective well-being, the effect of satisfaction derived from work life on their subjective well-being is greater compared to others. Conversely, for individuals with higher levels of subjective well-being, the contribution of work life satisfaction to their overall well-being is less significant. In other words, as the level of subjective well-being increases, the impact of satisfaction obtained from work life decreases. Literature shows that many studies have identified a positive relationship between income and subjective well-being [8,31,61,62,63]. In studies examining life satisfaction domains, variables such as income satisfaction, job satisfaction, and financial satisfaction have been incorporated into models, individually or in combination. In our study, however, a latent variable for work life satisfaction was created to represent satisfaction levels in work and income contexts. Both the national and international literature have established a positive effect of income satisfaction, job satisfaction, and financial satisfaction on happiness [24,25,26,27,28,29,64]. The results obtained from this study support the findings in the literature from a more specific perspective, demonstrating that, for individuals with lower subjective well-being, satisfaction derived from work life significantly and meaningfully enhances their levels of subjective well-being. This finding underscores how the level of subjective well-being shapes the impact of work satisfaction and highlights the importance of relationship satisfaction, particularly for individuals with lower levels of subjective well-being.

Social life satisfaction positively affects subjective well-being and exhibits a consistent relationship across different quantile levels. Whether at lower or higher quantiles, the effect of social life satisfaction on subjective well-being remains stable. In a study by Diener and Ryan [65], the authors discussed a positive relationship between high subjective well-being and sociability, suggesting a bidirectional causal link between these two variables. In other words, individuals with greater satisfaction in their social life tend to report higher subjective well-being, and those with higher subjective well-being are often more sociable. In previous studies, social satisfaction and leisure satisfaction have typically been evaluated as separate variables. For example, in their research on Türkiye, Güzel and Görmüş [28] and Soylu [64] found that social satisfaction positively affects both life satisfaction and happiness. However, Soylu [64] also noted that leisure satisfaction had no significant impact on life satisfaction. In contrast, Praag’s study using data from Germany showed that leisure satisfaction positively contributes to life satisfaction. These findings underscore the crucial role that social interactions and the quality of one’s social life play in sustaining and enhancing subjective well-being.

Relationship satisfaction also positively affects subjective well-being, and this effect varies according to quantile levels. For individuals with low subjective well-being, relationship satisfaction significantly enhances their well-being, whereas the effect is less pronounced for those with high subjective well-being. In other words, for individuals with high subjective well-being, satisfaction with relationships does not have a strong influence on their overall well-being. This is likely because individuals with high subjective well-being tend to socialize more easily and are more self-confident, allowing them to form their own social support systems with ease [65]. Therefore, it can be concluded that the effect of relationship satisfaction on subjective well-being is less significant for individuals with high subjective well-being compared to those with lower subjective well-being. Although the importance of relationship satisfaction may vary across cultures, studies in the literature have consistently shown that satisfaction with relationships (e.g., with friends and family) has a positive effect on happiness. Köksal, Uçak, and Şahin [26], similarly to the approach used in this study, identified relationship satisfaction as a single variable and found a positive and significant association between relationship satisfaction and happiness in Türkiye. These results, consistent with the findings of other studies in the literature, highlight the positive effect of relationships on subjective well-being. In addition to previous research, our findings provide more detailed insights, underscoring that for individuals with lower subjective well-being, the effect of relationship satisfaction is more pronounced. This emphasizes the crucial role that relationships play in enhancing well-being, particularly for those with initially lower levels of subjective well-being.

Satisfaction with public services has a positive effect on subjective well-being, and this effect decreases as the quantile levels increase. In other words, for individuals with higher levels of subjective well-being, satisfaction with public services has a smaller impact on their overall well-being. Similarly, Köksal, Uçak, and Şahin [26] incorporated all types of public services into a single variable, even though they did not treat public services satisfaction as a latent construct in their study. Their research on Türkiye demonstrated a positive relationship between public services satisfaction and happiness. However, due to the limited number of studies in the literature on this topic, these results need further research to be validated. Public services play a vital role in the quality of life by providing essential support and infrastructure that can enhance subjective well-being. The positive effect observed in this study suggests that effective public services contribute significantly to well-being, reinforcing the need for ongoing investment in public infrastructure and services.

This study’s limitations also present opportunities for future research. First, the data were collected solely from individuals in Türkiye, which limits the generalizability of the findings. This suggests the potential for conducting similar studies in different countries or cultural contexts to enhance the validity and reliability of the results. Secondly, the reliance on cross-sectional data from a single year may limit the ability to fully explore the causal relationships between subjective well-being and satisfaction domains. While longitudinal data would offer a more comprehensive understanding of the dynamic interactions between these variables, the application of such data within the framework of QSEM presents significant methodological challenges. The inclusion of multiple years, given the latent variable structure and the quantile-specific estimations employed in the analysis, would substantially increase the complexity of the model and potentially hinder interpretability. Therefore, this study’s focus on a single year was deemed appropriate to maintain the analytical clarity and focus of the findings. In addition, this study will provide a methodological reference for other studies in the field and may shed light on similar studies in the future. Lastly, the subjective well-being and satisfaction measurements used in the study are based on participants’ self-reports, which may be subject to biases such as social desirability and respondent tendencies. This limitation is common to all studies employing satisfaction domains based on self-reported data.

In addition, limitations are associated with the statistical methodology employed in this study. First, Bayesian prediction, while offering various advantages, entails certain challenges. Determining appropriate iteration counts and burn-in periods for convergence can require extensive experimentation, and, as the number of iterations grows—particularly in complex models—this process can become time-consuming, potentially leading to inefficiencies. Furthermore, compared to classical SEM, both the model’s overall fit assessment and parameter estimates are somewhat limited within the Bayesian framework. In particular, model fit in BQSEM can only be evaluated through the PPP criterion, which may not provide as comprehensive an assessment as in frequentist SEM approaches. Lastly, the application of QSEM in this study has been limited to linear models. Future research could explore extending QSEM to incorporate non-linear, non-parametric, or semi-parametric models among latent variables, expanding the applicability and robustness of the method in diverse contexts.

## 5. Conclusions

In conclusion, this study employs BSEM and BQSEM to provide valuable insights into the factors influencing individuals’ subjective well-being in Türkiye. These advanced methods, particularly the use of latent variable models and the innovative application of the quantile approach, offer a more nuanced understanding of how satisfaction in various life domains affects subjective well-being across different levels of well-being. This study is the first to apply BQSEM to subjective well-being, showcasing its potential to reveal the differential effects of predictors at various quantile levels.

These findings indicate that satisfaction with health, marriage, work life, social life, relationships, and public services all positively affect subjective well-being. Notably, for individuals with lower levels of subjective well-being, satisfaction with work life, relationships, and public services has a stronger effect on subjective well-being. In contrast, for those with higher levels of subjective well-being, marriage satisfaction affects subjective well-being more strongly. On the other hand, satisfaction with education and housing district did not show a significant effect, suggesting these areas may warrant further exploration.

These results emphasize that, when evaluating subjective well-being, it is crucial to consider not only economic indicators but also individuals’ personal and social satisfaction. Improving satisfaction levels in these domains can substantially improve overall life satisfaction. In summary, this study provides essential insights into the factors influencing subjective well-being in Türkiye and offers valuable guidance for enhancing overall quality of life through targeted improvements in these key areas.

## Figures and Tables

**Table 1:** Latent and observed variables utilized in the study.

Name of the Latent Variable	Name of the Observed Variable	Question
Subjective Well-Being	Happiness	1. When you think your life as a whole, how happy are you?
	Life Satisfaction	2. When you think as a whole, rate your recent life satisfaction between 0 and 10.
Housing-District Satisfaction	Housing	1. Are you satisfied with your residence houses?
	District	2. Are you satisfied with district where you live?
Work Life Satisfaction	Work	1. Are you satisfied with your job?
	Earning	2. Are you satisfied with the income that you get from your job?
	Income	3. Are you satisfied with your household monthly income?
	Time	4. Are you satisfied with the time spend (taking) for work from arrival to departure?
	Colleague	5. Are you satisfied with relationships with people about your business?
Social Life Satisfaction	Social life	1. Are you satisfied with social life (entertainment, cultural and sporting activities, etc.)?
	Personal Time	2. Are you satisfied with taking time for yourself?
Relationship Satisfaction	Relative	1. Are you satisfied with relationships with your relatives?
	Friend	2. Are you satisfied with relationships with your friends?
	Neighbor	3. Are you satisfied with relationships with your neighbors?
Public Service Satisfaction	Health	1. Are you satisfied with health services?
	Public order	2. Are you satisfied with the public security service?
	Judicial	3. Are you satisfied with judicial services?
	Education	4. Are you satisfied with the services of education?
	SSI	5. Are you satisfied with the services of Social Security Institution?
	Transportation	6. Are you satisfied with the services of transportation?
Health Satisfaction	-	Are you satisfied with your health?
Education Satisfaction	-	Are you satisfied with the education you have ever received?
Marriage Satisfaction	-	Are you satisfied with your marriage?

**Table 2:** Descriptive statistics and correlation coefficients of latent variables.

	M	SD	1	2	3	4	5	6
1. Subjective Well-Being	5.061	1.515	1					
2. Housing-District Satisfaction	3.815	0.648	0.262 **	1				
3. Work Life Satisfaction	3.489	0.629	0.536 **	0.340 **	1			
4. Social Life Satisfaction	3.140	0.925	0.447 **	0.270 **	0.547 **	1		
5. Relationship Satisfaction	3.841	0.565	0.301 **	0.312 **	0.321 **	0.308 **	1	
6. Public Service Satisfaction	3.652	0.612	0.380 **	0.297 **	0.405 **	0.384 **	0.347 **	1

Note: M = mean; SD = standard deviation. ** *p* < 0.001.

**Table 3:** Estimated parameter coefficients and credible intervals at a 95% confidence level for BSEM and Median-BQSEM.

	Parameters	BSEM	Credible İnterval	Median BQSEM	Credible İnterval
Factor loads	λ_21_	0.924	[0.865; 0.985]	0.933	[0.89; 0.982]
	λ_42_	0.834	[0.728; 0.943]	0.940	[0.890; 0.990]
	λ_63_	1.513	[1.429; 1.603]	2.662	[2.470; 2.803]
	λ_73_	1.492	[1.408; 1.582]	2.694	[2.499; 2.838]
	λ_83_	0.728	[0.656; 0.804]	1.528	[1.404; 1.636]
	λ_93_	0.521	[0.448; 0.595]	0.404	[0.363; 0.441]
	λ_11,4_	0.887	[0.831; 0.943]	0.978	[0.950; 1.006]
	λ_13,5_	1.057	[0.981; 1.140]	1.057	[1.013; 1.101]
	λ_14,5_	0.872	[0.799; 0.951]	1.034	[0.996; 1.072]
	λ_16,6_	0.990	[0.927; 1.058]	0.649	[0.608; 0.693]
	λ_17,6_	1.025	[0.958; 1.094]	1.112	[1.076; 1.15]
	λ_18,6_	0.987	[0.922; 1.056]	1.218	[1.170; 1.270]
	λ_19,6_	0.977	[0.915; 1.044]	1.126	[1.09; 1.161]
	λ_20,6_	0.742	[0.678; 0.806]	1.044	[1.004; 1.082]
Structural Model Coefficients	b_0_—Constant	0.000	-	*−0.007*	[−0.034; 0.018]
	b_1_—Health Satisfaction	0.106	[0.075; 0.136]	0.113	[0.081; 0.145]
	b_2_—Education Satisfaction	*0.009*	[−0.022; 0.039]	*0.028*	[−0.001; 0.057]
	b_3_—Marriage Satisfaction	0.104	[0.075; 0.133]	0.110	[0.079; 0.138]
	γ_1_—Housing-District Satisfaction	*−0.014*	[−0.082; 0.054]	*0.017*	[−0.046; 0.086]
	γ_2_—Work Life Satisfaction	0.540	[0.460; 0.622]	0.862	[0.740; 0.987]
	γ_3_—Social Life Satisfaction	0.192	[0.135; 0.251]	0.221	[0.173; 0.269]
	γ_4_—Relationships Satisfaction	0.126	[0.064; 0.189]	0.106	[0.042; 0.169]
	γ_5_—Public Service Satisfaction	0.168	[0.109; 0.228]	0.170	[0.109; 0.227]
Covariance between Latent Variables	ϕ_11_	0.524	[0.451; 0.608]	0.426	[0.381; 0.472]
	ϕ_12_	0.175	[0.149; 0.203]	0.078	[0.066; 0.092]
	ϕ_13_	0.236	[0.200; 0.275]	0.200	[0.169; 0.232]
	ϕ_14_	0.214	[0.182; 0.247]	0.199	[0.172; 0.225]
	ϕ_15_	0.199	[0.170; 0.230]	0.165	[0.141; 0.189]
	ϕ_22_	0.349	[0.309; 0.391]	0.128	[0.114; 0.148]
	ϕ_23_	0.305	[0.274; 0.338]	0.177	[0.159; 0.198]
	ϕ_24_	0.120	[0.098; 0.143]	0.058	[0.046; 0.070]
	ϕ_25_	0.162	[0.140; 0.186]	0.080	[0.069; 0.093]
	ϕ_33_	0.728	[0.663; 0.793]	0.773	[0.722; 0.829]
	ϕ_34_	0.237	[0.204; 0.275]	0.203	[0.173; 0.235]
	ϕ_35_	0.265	[0.232; 0.298]	0.235	[0.207; 0.265]
	ϕ_44_	0.488	[0.432; 0.546]	0.465	[0.428; 0.504]
	ϕ_45_	0.203	[0.175; 0.232]	0.188	[0.164; 0.212]
	ϕ_55_	0.477	[0.429; 0.528]	0.438	[0.404; 0.476]

Note: Those that are statistically insignificant at 95% confidence level are shown in *italics*.

**Table 4:** Estimated parameter coefficients for BSEM and BQSEM.

	BSEM	BQSEM						
Parameters	-	5%	10%	25%	50%	75%	90%	95%
Structural Model Coefficients								
b_0_—Constant	0.000	−0.520	−0.446	−0.316	*−0.007*	0.298	0.440	0.505
b_1_—Health Satisfaction	0.106	0.110	0.113	0.111	0.113	0.114	0.115	0.118
b_2_—Education Satisfaction	*0.009*	*0.019*	*0.020*	*0.024*	*0.028*	*0.023*	*0.023*	*0.019*
b_3_—Marriage Satisfaction	0.104	0.094	0.097	0.098	0.110	0.131	0.131	0.138
γ_1_—Housing-District Satisfaction	*−0.014*	*−0.010*	*0.002*	*−0.002*	*0.017*	*0.033*	*0.032*	*0.033*
γ_2_—Work Life Satisfaction	0.540	0.919	0.898	0.896	0.862	0.772	0.733	0.748
γ_3_—Social Life Satisfaction	0.192	0.238	0.234	0.234	0.221	0.220	0.228	0.226
γ_4_—Relationships Satisfaction	0.126	0.145	0.137	0.135	0.106	0.091	0.091	0.081
γ_5_—Public Service Satisfaction	0.168	0.200	0.186	0.185	0.170	0.139	0.130	0.136

Note: Those that are statistically insignificant at 95% confidence level are shown in *italics*.

## Data Availability

Due to legal or ethical reasons, the data used in the study cannot be shared as it was obtained from the Turkish Statistical Institute.
